# The Viral Polymerase Complex Mediates the Interaction of Viral Ribonucleoprotein Complexes with Recycling Endosomes during Sendai Virus Assembly

**DOI:** 10.1128/mBio.02028-20

**Published:** 2020-08-25

**Authors:** Emmanuelle Genoyer, Katarzyna Kulej, Chuan Tien Hung, Patricia A. Thibault, Kristopher Azarm, Toru Takimoto, Benjamin A. Garcia, Benhur Lee, Seema Lakdawala, Matthew D. Weitzman, Carolina B. López

**Affiliations:** a Department of Pathobiology, School of Veterinary Medicine, University of Pennsylvania, Philadelphia, Pennsylvania, USA; b Division of Protective Immunity and Division of Cancer Pathobiology, The Children’s Hospital of Philadelphia, Philadelphia, Pennsylvania, USA; c Department of Pathology and Laboratory Medicine, Perelman School of Medicine, University of Pennsylvania, Philadelphia, Pennsylvania, USA; d Department of Microbiology, Icahn School of Medicine at Mount Sinai, New York, New York, USA; e Department of Microbiology and Immunology, University of Rochester Medical Center, Rochester, New York, USA; f Department of Biochemistry and Biophysics, Perelman School of Medicine, University of Pennsylvania, Philadelphia, Pennsylvania, USA; g Epigenetics Institute, Perelman School of Medicine, University of Pennsylvania, Philadelphia, Pennsylvania, USA; h Department of Microbiology & Molecular Genetics, University of Pittsburgh School of Medicine, Pittsburgh, Pennsylvania, USA; Columbia University Medical College

**Keywords:** paramyxovirus, recycling endosomes, virus assembly

## Abstract

Paramyxoviruses are negative-sense single-stranded RNA viruses that comprise many important human and animal pathogens, including human parainfluenza viruses. These viruses bud from the plasma membrane of infected cells after the viral ribonucleoprotein complex (vRNP) is transported from the cytoplasm to the cell membrane via Rab11a-marked recycling endosomes. The viral proteins that are critical for mediating this important initial step in viral assembly are unknown. Here, we used the model paramyxovirus, murine parainfluenza virus 1, or Sendai virus (SeV), to investigate the roles of viral proteins in Rab11a-driven virion assembly. We previously reported that infection with SeV containing high levels of copy-back defective viral genomes (DVGs) (DVG-high SeV) generates heterogenous populations of cells. Cells enriched in full-length (FL) virus produce viral particles containing standard or defective viral genomes, while cells enriched in DVGs do not, despite high levels of defective viral genome replication. Here, we took advantage of this heterogenous cell phenotype to identify proteins that mediate interaction of vRNPs with Rab11a. We examined the roles of matrix protein and nucleoprotein and determined that their presence is not sufficient to drive interaction of vRNPs with recycling endosomes. Using a combination of mass spectrometry and comparative analyses of protein abundance and localization in DVG-high and FL-virus-high (FL-high) cells, we identified viral polymerase complex component protein L and, specifically, its cofactor C as interactors with Rab11a. We found that accumulation of L and C proteins within the cell is the defining feature that differentiates cells that proceed to viral egress from cells containing viruses that remain in replication phases.

## Abstract (precis)

Paramyxoviruses are members of a family of viruses that include a number of pathogens imposing significant burdens on human health. In particular, human parainfluenza viruses are an important cause of pneumonia and bronchiolitis in children for which there are no vaccines or directly acting antivirals. These cytoplasmic replicating viruses bud from the plasma membrane and co-opt cellular endosomal recycling pathways to traffic viral ribonucleoprotein complexes from the cytoplasm to the membrane of infected cells. The viral proteins required for viral engagement with the recycling endosome pathway are still not known. Here, we used the model paramyxovirus Sendai virus, or murine parainfluenza virus 1, to investigate the role of viral proteins in this initial step of viral assembly. We found that the viral polymerase components large protein L and accessory protein C are necessary for engagement with recycling endosomes. These findings are important in identifying viral proteins as potential targets for development of antivirals.

## INTRODUCTION

Paramyxoviruses are single-stranded negative-sense RNA viruses that include viruses of clinical and global health significance, such as human parainfluenza viruses (HPIV). HPIV1 and HPIV2 are the leading causes of croup in young children, and HPIV3 is associated with bronchiolitis, bronchitis, and pneumonia (1). These infections have also been associated with development or exacerbation of asthma and chronic airway disorders (2). Additionally, HPIVs are the etiological agents of 7% of hospitalized pneumonia cases in children in the United States (3), as well as in Africa and Asia (4). Although HPIVs impose a significant health burden, there are no directly acting antivirals or preventative vaccines. In order to identify targets for antiviral development, there is a need to better understand fundamental aspects of paramyxovirus biology, including mechanisms of virion assembly and particle production.

Sendai virus (SeV), or murine parainfluenza virus 1, is closely related to human parainfluenza viruses 1 and 3, with 69% homology at the RNA level between SeV and HPIV1. SeV has long served as a well-established model for understanding paramyxovirus replication. SeV genome replication occurs in the cytoplasm of infected cells (5), and virions bud from the plasma membrane. The viral genomes are tightly coated in the viral nucleocapsid protein (NP), with one NP molecule for every six nucleotides of genome (6). This viral RNA (vRNA)-protein complex is referred to as the viral ribonucleoprotein (vRNP) (7, 8). Viral genomes are replicated by the RNA-dependent RNA polymerase (RdRP) large protein, or protein L (9), along with the polymerase cofactor phosphoprotein (P), which is required for recruitment of NP to nascent RNA (10). Additionally, many paramyxoviruses encode a family of accessory proteins known as C proteins (C′, C, Y1, and Y2) generated by translation of the P mRNA in an alternative reading frame. The C proteins were first described as interferon antagonists via binding to STAT1 (11) but have also been implicated as polymerase cofactors that regulate genome polarity and mRNA transcription (12).

The viral structural proteins include the hemagglutinin (HN) and fusion (F) surface proteins, which act as an attachment factor and fusion machinery, respectively (13). The matrix protein (M) lines the inner membrane of the virion and is important for bridging the interactions between HN, F, and vRNP complexes, and these interactions drive the membrane curvature process, which results in the budding of virions (14, 15). In order for virion release to occur at the membrane, cytoplasmic vRNPs and structural proteins must be transported to the cell surface. While F and HN are trafficked and processed through the endosomal network to the plasma membrane (16), vRNPs require transport through the cytoplasm.

Paramyxoviruses, as well as orthomyxoviruses and hantaviruses, use the recycling endosome pathway as a controlled mechanism of egress to traffic vRNPs from the infected cell cytoplasm to the cell membrane (17). Rab11a, a host GTPase associated with recycling endosomes, catalyzes bidirectional transport of endosomes along microtubule networks between the perinuclear endocytic recycling complex and the plasma membrane (18). The paramyxoviruses that rely on Rab11a for intracellular transport and viral egress include SeV (19), measles virus (20), mumps virus (21), and HPIV1 (19). While it is known that the vRNP interacts with Rab11a, it is not known which viral proteins are necessary to drive this critical interaction required for viral particle assembly. In fact, although multiple proteins, including M and C, have been investigated for their role in driving viral assembly, their roles remain contested and poorly understood (14). For example, SeV M has been reported to interact with vRNPs in the cytoplasm and to be required for recruitment of vRNPs to the membrane (22, 23) but has also been shown to traffic with surface proteins F and HN via the trans-Golgi network (TGN) (24). Whether the M protein is critical for engagement of SeV vRNPs with Rab11a-marked endosomes is unknown. Further, in addition to the roles of the C proteins in innate immune antagonism and polymerase function, they have also been reported to be enhancers of particle formation (25, 26). Data suggest that C is critical in recruiting vRNPs to the membrane (25) and that C expression enhances associations of vRNPs with membranes. Additionally, both C and M proteins have been shown independently to recruit Aip1/Alix, a protein involved in endosomal sorting and vesicle budding, to the plasma membrane to enhance virion formation (26, 27). However, the hypothesis of the requirement for Aip1/Alix in paramyxovirus budding is contested, with evidence suggesting that absence of Aip1/Alix in cells does not have any effect on SeV particle formation (28). Thus, while both C and M have been heavily studied in the context of particle assembly, a clear picture of how these proteins function in early steps of assembly is lacking.

**FIG 1:**
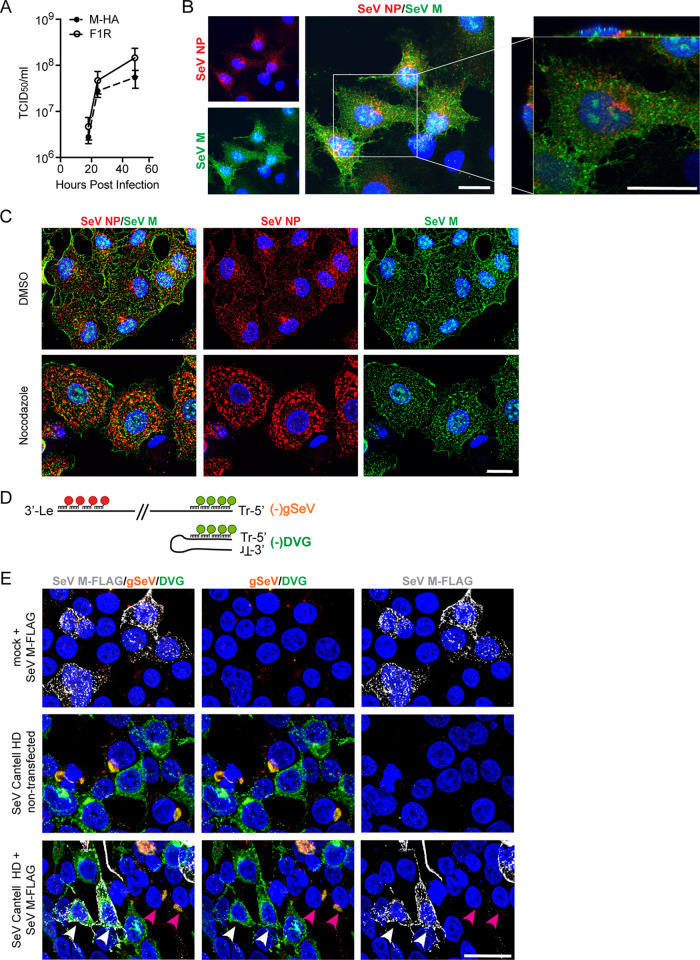
Matrix protein localization to the plasma membrane occurs independently of microtubules/Rab11a. (A) TCID_50_/ml of supernatant from LLCMK2 cells infected with SeV-M-HA and SeV F1R (parental wild type) at the indicated time point, *n* = 3, graphed as means ± standard errors of the means (SEM). (B) A549 cells infected with SeV-M-HA for 24 hpi; immunofluorescence for SeV NP (red), HA (SeV M, green), Hoechst (nuclei, blue). Confocal 63×, 2× digital zoom images are shown. The three panels at left show extended-focus images; the panel at right shows cropped single-*xy* plane as well as *xz* and *yz* plane images. (C) A549 cells infected with SeV-M-HA, treated with nocodazole or dimethyl sulfoxide (DMSO) vehicle control at 4 hpi; immunofluorescence at 24 hpi for SeV NP (red), HA (SeV M, green), and Hoechst (nuclei, blue). A 63× widefield deconvolved, maximum projection image is shown. (D) Schematic of vRNA FISH to detect (-)gSeV and (-)DVG RNA, indicating red and green probe binding regions. (E) 293T cells infected with SeV Cantell HD and then transfected with SeV M-FLAG at 6 hpi; vRNA FISH with immunofluorescence for FLAG (SeV M, gray) at 24 hpi. A 63× widefield deconvolved, maximum projection image is shown. White arrows indicate transfected and infected DVG-high cells, and magenta arrows indicate FL-high cells. All images are representative of results from 3 independent experiments; scale bar = 20 μm.

Defective viral genomes (DVGs) are truncated products generated during viral replication that are able to be replicated only in the presence of a full-length (FL) standard viral genome (29). DVGs of the copy-back type (cbDVGs) are robustly generated during infection with paramyxoviruses, including measles virus (30), mumps virus (31), Nipah virus (32), and SeV (33, 34). cbDVGs are generated from the 5′ end of the antigenome when the viral polymerase detaches from the template strand and resumes copying the nascent strand, leading to a theoretical hairpin loop with complementary ends (Fig. 1D). DVGs have been previously described to alter outcomes of infection by reducing standard viral replication, initiating viral persistence, and inducing innate immune responses (29, 35). In addition to playing a critical role in modulating viral infections, DVGs have long been used as a tool for dissecting basic processes of viral replication (6, 36), innate immune activation (37, 38), and particle production (39).

We previously reported that upon infection with DVG-containing virus populations, cells display a heterogenous phenotype with the development of subpopulations of virus containing DVG-high cells and full-length (FL)-high cells (40, 41). DVG-high cells contain higher levels of DVGs than of full-length genomes, and FL-high cells contain higher levels of full-length genomes than of DVGs. These subpopulations not only have distinct transcriptional profiles (40), but they also have different intracellular localizations of vRNA (41). The vRNA in FL-high cells interacts with recycling endosomes, and this leads to the production of both standard and defective viral particles. In contrast, the vRNA in DVG-high cells does not interact with recycling endosomes; consequently, these cells do not produce significant amounts of viral particles. These DVG-high cells do, however, undergo robust levels of vRNA replication, as evidenced by the large increase in DVG RNA revealed by qPCR and vRNA fluorescent *in situ* hybridization (FISH) over time (40, 41). Here, we took advantage of DVGs as a system to investigate the initial steps that differentiate viral replication from viral particle production, namely, how vRNPs interact with Rab11a. We describe viral polymerase components L and C as differentiating factors in FL-high cells that facilitate vRNP association with recycling endosomes and subsequent viral assembly.

## RESULTS

### M protein interacts with NP primarily at the cell surface and does not localize with Rab11a.

In order to investigate whether the M protein is responsible for the association of vRNPs with recycling endosomes, we created a recombinant SeV with a hemagglutinin (HA) tag on the N terminus of the M protein (SeV-M-HA) to study its localization during infection. We characterized this virus to ensure that the HA tag did not result in a growth curve dramatically different from that seen with the parental SeV F1R strain (SeV-F1R). We found that while viral output was slightly lower at later time points in infection, virion production was largely unimpaired (Fig. 1A). We then examined the localization of M during infection. Consistent with the fact that M lines the inner side of virions and budding occurs from the plasma membrane, we observed M at the plasma membrane of infected cells (Fig. 1B and C). Interestingly, single-plane confocal images showed little overlap of NP and M proteins (Fig. 1B). This virus allows us to define M protein intracellular distribution during replication and virion assembly.

As we previously reported, when Rab11a is knocked down by small interfering RNA (siRNA) or when microtubule polymerization is disrupted, the perinuclear localization of viral RNA is altered (41). To ask if M interacted with the Rab11a/microtubule pathway, we assayed localization of M upon treatment with nocodazole, a drug that prevents microtubule polymerization. In agreement with previously published data, nocodazole treatment of FL-high cells disrupted perinuclear clustering of the viral NP, indicating that vRNPs are tethered to microtubules via recycling endosomes (41). In contrast, M protein distribution was not drastically altered when cells were treated with nocodazole and it still localized at the membrane (Fig. 1C). These data support a model whereby the M protein is trafficked to the cell membrane independently of the microtubule network, implying that the M protein is unlikely to be critical in driving interactions between vRNPs and recycling endosomes.

It has also been previously reported that the presence of DVGs leads to increased degradation and turnover of M (42). Therefore, if M is the protein responsible for tethering vRNPs to recycling endosomes, it is possible that DVGs in DVG-high cells fail to interact with Rab11a due to insufficient levels of M to drive this interaction. To address this possibility, we overexpressed M-FLAG in cells infected with SeV strain Cantell with a high level of DVGs (Cantell HD), which generated a heterogenous population of DVG-high and FL-high cells (40, 41), and asked whether high levels of M were sufficient to drive a perinuclear localization of DVGs. For these experiments, we used 293T cells because they allow infection and subsequent transfection of the same cell. We used vRNA FISH to distinguish DVG from genomic SeV (gSeV) RNA (Fig. 1D) and confirmed that the distinct intracellular distributions of FL-high and DVG-high cells were conserved in these cells. Overexpressed M localized to the membrane of infected cells, as expected. However, overexpression of M in DVG-high cells failed to recruit DVGs to the perinuclear region, with DVGs remaining distributed throughout the host cell cytoplasm (Fig. 1E). These data indicate that the presence of M is not sufficient to drive association between vRNPs and recycling endosomes.

**FIG 2:**
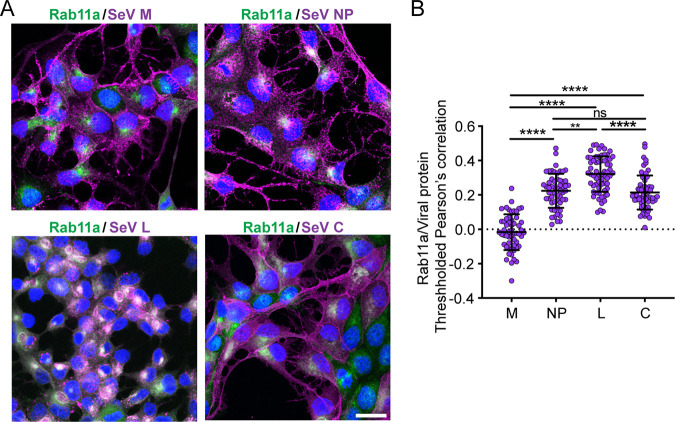
vRNP components colocalize with Rab11a, but SeV M does not. (A) A549-Rab11a-mCherry cells infected with SeV M-HA (M and NP), SeV Cantell LD (C), or SeV LeGFP (L) for 24 h. Images show immunofluorescence detection of viral antigens (purple) and mCherry-Rab11a (green). Confocal 63× images (1.5× zoom, maximum projection) are shown. All images are representative of results from 3 independent experiments; scale bar = 20 μm. (B) Quantification of colocalizations between viral proteins and Rab11a, pooled from three independent experiments with >20 cells analyzed per experiment. Individual cells are plotted with the horizontal dotted line at the mean; error bars represent standard deviations (SD). **** = *P* < 0.0001; ** = *P* < 0.01 (by one-way analysis of variance [ANOVA] with Sidak’s multiple-comparison test); ns, not significant.

Finally, to confirm that M was not responsible for driving interactions between vRNPs and recycling endosomes, we infected A549 Rab11a-mCherry cells with SeV low-DVG (LD) viruses, in which all vRNPs should interact with Rab11a (41). We performed immunofluorescence for various viral proteins (shown in magenta in Fig. 2A) and compared the results with respect to colocalization with Rab11a (pseudocolored green; Fig. 2B). In SeV LD infections, NP and Rab11a colocalize in the perinuclear region, as previously reported (41). In contrast, M largely does not colocalize with Rab11a (Fig. 2B). We also examined colocalization of Rab11a with other viral proteins, including C proteins and polymerase L proteins, using a previously characterized strain with the L protein C-terminally fused to enhanced green fluorescent protein (SeV LeGFP) (43) to visualize L protein during infection. We found that C also colocalizes with Rab11a to the same extent as NP and that L colocalizes to an even higher degree than NP or C (Fig. 2B). These data indicate that at this time point of infection, most or all of the L protein is associated with recycling endosomes. Overall, these data support a model whereby vRNPs associate with Rab11a independently of M and likely through vRNP components such as NP, L, or C.

### Nucleoprotein coverage of vRNPs is not sufficient to drive interaction with Rab11a.

**FIG 3:**
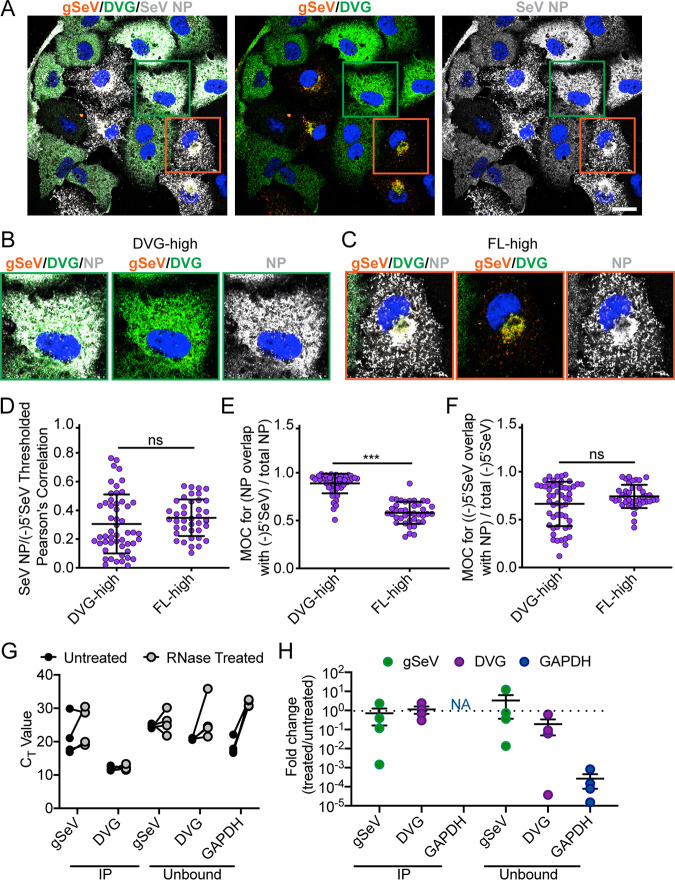
Nucleoprotein associations with full-length and defective viral genomes are similar. (A) A549 cells infected with SeV Cantell HD and then subjected to RNA FISH with immunofluorescence for NP (gray) and Hoechst staining (nuclei, blue) detected at 24 hpi. Confocal 63×, 1.5× zoom, single-plane images are shown. Images are representative of results from three independent experiments; scale bar = 20 μm. The green boxes indicate representative DVG-high cells, and the orange boxes indicate representative FL-high cells. (B) Representative cropped DVG-high cell. (C) Representative cropped FL-high cell. (D) Costes’s Pearson’s correlation of colocalization between (-)5′ SeV and SeV NP in individual DVG-high and FL-high cells. (E) Mander’s overlap coefficient (MOC) of NP with 5′ SeV in DVG-high and FL-high cells. (F) Mander’s overlap coefficient (MOC) of 5′ SeV with NP in DVG-high and FL-high cells. Individual cells pooled from three independent experiments are plotted with the horizontal dotted line at the mean; error bars represent SD. **** = *P* < 0.0001 by Mann-Whitney U-test. (G) *C_T_* values for qPCR for viral RNA, DVG RNA, and GAPDH with and without RNase A, V1, and T1 treatment after immunoprecipitation with anti-SeV NP. (H) Fold change in RNA levels in RNase-treated immunoprecipitation products relative to those left untreated. Data from four independent experiments are shown with the horizontal dotted line at the mean; error bars represent SEM.

Since we observed that NP, L, and C proteins all colocalized with Rab11a during infection, we next sought to address which of these proteins is important for the interaction of vRNPs with recycling endosomes. First, we asked whether NP is sufficient for driving this interaction. As the most abundant protein on the vRNP, it is possible that NP coverage of the viral genome dictates engagement with Rab11a. Viral RNA in DVG-high cells does not interact with Rab11a (41); therefore, if NP were critical for interacting with Rab11a, viral RNA in DVG-high cells would be uncoated or only partially coated in NP. If viral RNA in DVG-high cells were found to be coated in NP, then we could conclude that the presence of NP was not sufficient to drive the interaction between viral RNA and Rab11a. In order to investigate whether viral RNA in DVG-high cells was coated with NP, we tested for colocalization of these NP and viral genomes using RNA FISH combined with immunofluorescence for NP (Fig. 3A). As previously reported, upon infection with SeV HD, we saw the accumulation of DVG-high and FL-high cells. These cells have discrete intracellular localizations of viral RNA and viral NP as well as various levels of nucleoproteins as reported and quantified previously (41). In order to investigate whether the differences in the amounts or localizations of viral NP affected its interaction with viral RNA, we quantified colocalization of 5′(-)SeV RNA with SeV NP within the two distinct cellular subsets (Fig. 3D to F). As shown in Fig. 1D, the 5′(-)SeV probe set bound to all species of negative-sense vRNA in cells. The majority of vRNA recognized by 5′(-)SeV probe RNA corresponded to DVGs in DVG-high cells (Fig. 3B), but the majority corresponded primarily to FL-genome RNA in FL-high cells, as indicated in Fig. 3C. We found that there were no significant differences in colocalization of viral RNA and SeV NP between DVG-high and FL-high cells (Fig. 3D). Mander’s overlap coefficient (MOC) indicates the portion of a signal that overlaps the other signal in question, regardless of signal intensity. The MOC for NP with viral RNA showed that nearly all NP in DVG-high cells overlapped viral genomes but that a lower fraction of SeV NP overlapped vRNA in FL-high cells, presumably due to excess free NP that was not coating viral genomes (Fig. 3E). The MOC results for 5′(-)SeV RNA overlap NP were equivalent between DVG-high and FL-high cells, indicating that equal proportions of viral RNA colocalized with NP (Fig. 3F).

Though DVGs on the whole appear to be associated with NP, we wondered whether the NP coating of DVGs was complete. We reasoned that there might be regions of exposed RNA that might disrupt the helical structure of the vRNP and therefore prevent interactions with host factors. To assess the integrity of the vRNP, we performed immunoprecipitation (IP) of NP from infected cells and subjected the IP product to RNase digestion with a combination of RNase A, V, and T1, targeting both single-stranded and double-stranded RNA. RNA was then extracted and analyzed by real-time quantitative PCR (RT-qPCR) to compare levels of viral RNA to the levels seen with untreated controls. Reverse transcription was primed distal to qPCR primer sites and close to the 5′ end of the genome to capture degradation at any site across the vRNA. There were no significant differences in RNA quantities between DVG RNA and full-length vRNA under treated and untreated conditions (Fig. 3G). We also tested unbound fractions of the pulldown, reasoning that if any free RNA were present it would not be precipitated by anti-NP antibody (Ab). Both gSeV RNA and DVG RNA were found in the unbound fraction, indicating that the IP did not precipitate all of the vRNPs. The RNAs in this fraction also did not significantly differ between the treated and untreated conditions, particularly in comparison to an abundant cellular mRNA, GAPDH (glyceraldehyde-3-phosphate dehydrogenase) (Fig. 3H). These data indicate that the vast majority of vRNPs present during high-DVG infections are sufficiently coated in NP. Our results indicate that whereas NP-coated vRNAs associate with Rab11a in FL-high cells, NP coating of viral genomes is not sufficient to drive association with recycling endosomes, as evidenced by the presence of NP on vRNA in DVG-high cells that does not interact with Rab11a. It is therefore likely that a component of the vRNP complex other than NP is providing a link between viral RNA and Rab11a.

### DVG-driven interference leads to strong decreases in levels of L transcripts and protein.

Accumulation of DVGs interferes with standard vRNA replication and leads to a decrease in viral protein accumulation. This interference is due to DVGs competing for viral polymerase and other proteins necessary for vRNA replication (44, 45). In order to employ a more systematic approach to examine the role of viral proteins in viral assembly, we investigated whether the interference effect resulting from high DVG levels has equal impacts across viral proteins or whether certain viral mRNAs and proteins are more significantly impacted by interference. We hypothesized that the proteins most extensively interfered with by DVGs are essential for the localization of vRNPs to recycling endosomes and therefore that vRNPs mislocalize within the cytoplasm in their absence.

**FIG 4:**
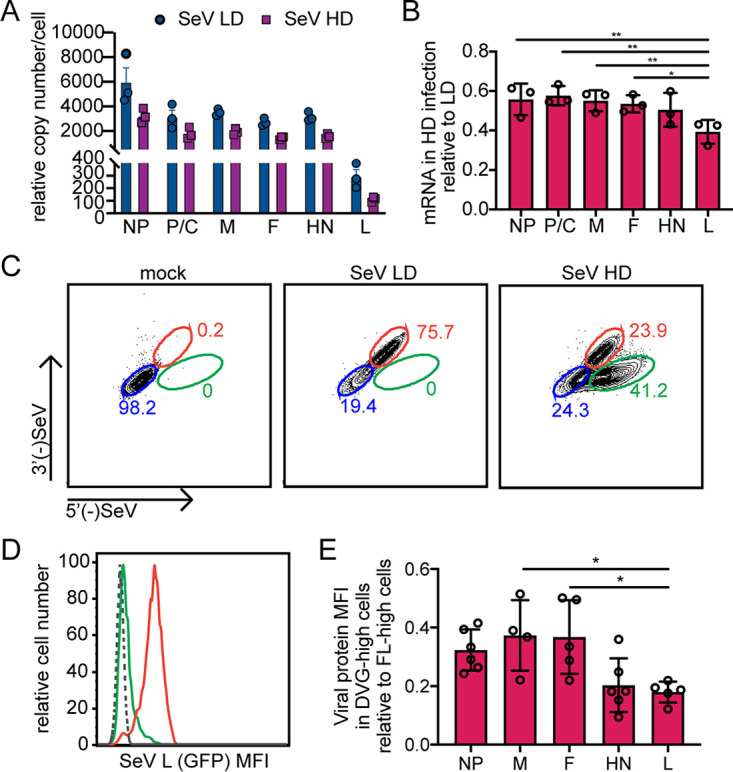
L mRNA and protein levels are significantly reduced in DVG-high cells compared to those of other viral proteins. (A and B) A549 cells infected with SeV Cantell LD and SeV Cantell HD for 12 h, qPCR for viral genes shown as relative copy number compared to GAPDH (A), and relative amount in SeV HD infections compared to SeV LD infections for each viral gene (B). ** = *P* < 0.01; * = *P* < 0.05 (by one-way ANOVA with Sidak’s multiple-comparison test). (C to E) A549 cells infected with SeV Cantell LD and SeV Cantell HD were subjected to RNA FISH combined with antibody staining for flow cytometry at 24 hpi. (C) Representative flow plots for each condition with gates for DVG-high cells highlighted in green, FL-high cells in red, and cells below limit of detection for viral RNA by flow in blue. (D) Representative mean fluorescence intensity (MFI) plot showing MFI of viral proteins in different cell populations in HD-infected cells with FL-high cells shown in red and DVG-high cells shown in green; mock cells shown with dashed line. (E) Relative MFI levels in DVG-high cells compared to FL-high cells under SeV HD-infected conditions; results of four independent experiments are shown, with bars representing means and SD. NP, F, and HN were detected with monoclonal antibodies during SeV Cantell HD infection. M was detected by HA during SeV-M-HA infection, and L was detected by GFP during SeV LeGFP infection in the presence of purified defective particles. * = *P* < 0.05 (by one-way ANOVA with Sidak’s multiple-comparison test).

The transcription of paramyxovirus mRNA is directed by the RdRP, which uses a stop-start mechanism. As the viral polymerase pauses at intergenic regions, some portion of the polymerases fall off prior to reinitiation, leading to a polar gradient of mRNA, with the highest levels of the 3′ proximal genes and the lowest levels of the 5′ genes produced (46, 47). To determine whether interference with mRNA transcription by DVGs had a greater effect on some viral mRNAs than on others, we performed qPCR on viral RNA from SeV LD-infected and HD-infected cell populations at 12 h postinfection (hpi), the time at which there is the highest rate of mRNA accumulation compared to genome accumulation (12, 41). As expected, we observed a gradient of transcription, with higher levels of the 3′ proximal transcripts and smaller amounts of L mRNA. Additionally, SeV HD infections showed lower levels of viral mRNA levels across all genes (Fig. 4A). However, comparing the levels of each mRNA across infections to normalize for differences in mRNA levels, it became clear that the levels of L mRNA were the most significantly decreased (Fig. 4B). Because the SeV HD infections include both FL-high and DVG-high cells, it is likely that the levels of mRNA in the DVG-high cells were even further reduced compared to the levels of viral mRNA in the LD infections, which contained mostly FL-high cells.

To determine whether decreases in viral mRNA transcription translate to lower levels of viral protein, we quantified protein levels in DVG-high and FL-high cells. SeV HD infections were subjected to RNA FISH-flow cytometry coupled with immunostaining for different viral proteins. RNA FISH-flow cytometry enables identification of discrete cell populations based on the intensities of 3′ and 5′ SeV probes in each cell, using the same strategy as was used for imaging (Fig. 1D). Using this method, we were able to define DVG-high and FL-high cell populations as present within the same infection (Fig. 4C) as validated previously (40). We also assayed levels of viral proteins in DVG-high and FL-high cell populations (Fig. 4D and E). Levels of protein in DVG-high cells were normalized to FL-high cell levels (Fig. 4E). Levels of all viral proteins were lower in the DVG-high cells than in the FL-high cells, conforming to the mRNA data. These data show that the proteins that were the least abundant were the most sensitive to DVG-mediated interference, since there was a much greater reduction of L protein levels in DVG-high cells than of other viral proteins (Fig. 4E). This observation suggests that the L protein may be an important factor in regulating interactions of vRNPs with recycling endosomes, as this is the factor that most strongly differentiates between DVG-high and FL-high cells.

### SeV C proteins interact with Rab11a.

**FIG 5:**
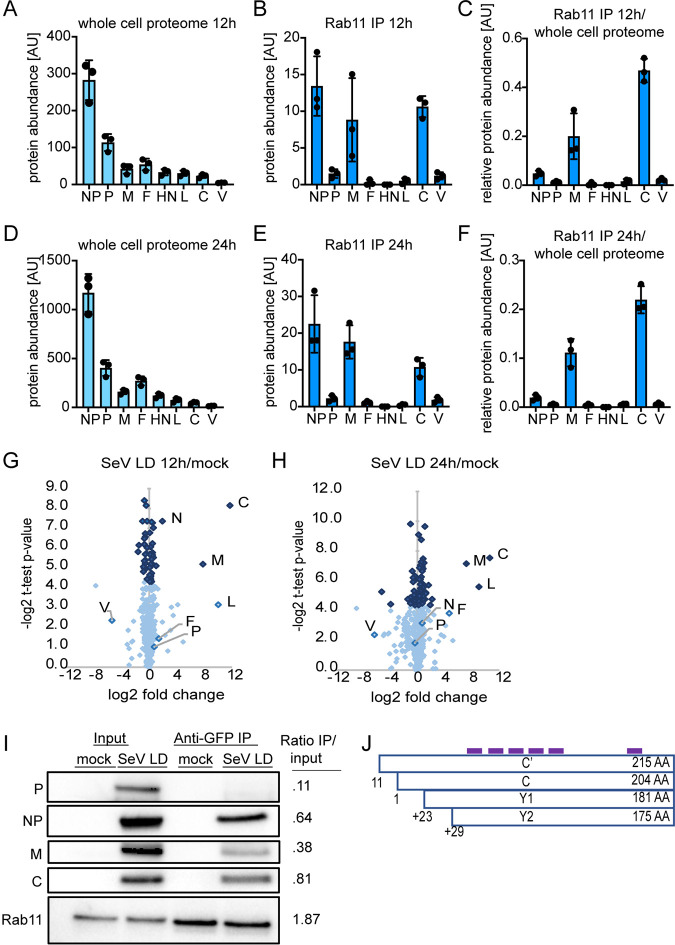
C proteins identified to interact with Rab11a by immunoprecipitation and mass spectrometry. (A) Viral protein abundance identified by mass spectrometry in the whole-cell lysate at 12 hpi. AU, arbitrary units. (B) Viral protein abundance identified by IP of Rab11a-GFP at 12 hpi. (C) Relative ratio of IP/whole-cell lysate of viral proteins at 12 hpi. (D) Viral protein abundance identified by mass spectrometry in the whole-cell lysate at 24 hpi. (E) Viral protein abundance identified by IP of Rab11a-GFP at 24 hpi. (F) Relative ratio of IP/whole-cell lysate of viral proteins at 24 hpi. In panels A to F, results of three independent experiments are plotted, with bars representing mean and error bars representing ± SD. (G and H) Volcano plot highlighting proteins enriched in Rab11a-GFP interacting protein at 12 hpi (G) and at 24 hpi (H) compared to mock treatment results. The abundance of each protein identified in IP was normalized by its abundance identified in the whole-cell lysate. In order to estimate an enrichment of proteins detected only under mock or infection conditions, missing values were imputed. Data are pooled from three independent experiments. Viral proteins are indicated. Dark blue dots represent *P* values of <0.05, and light blue dots represent *P* values of >0.05 (Student’s *t* test). (I) Validation of mass spectrometry by Western blotting probing for Rab11a or viral proteins at 24 hpi and quantified for relative band intensity of IP over input. (J) Schematic of C proteins, with locations of peptides identified by mass spectrometry in IP samples marked in purple.

We next used an unbiased approach to determine which viral proteins interact with Rab11a. To do this, we infected Rab11a-GFP A549 cells with SeV LD followed by immunoprecipitation (IP) of Rab11a using antibodies against green fluorescent protein (GFP) at 12 and 24 hpi to identify interacting proteins (see Table S1 in the supplemental material). By using SeV LD virus stocks, we ensured that all cells had vRNPs that were interacting with Rab11a. We also performed mass spectrometry on whole-cell lysates in order to obtain total quantification of all viral protein levels (Table S2). We identified all eight viral proteins in the whole-cell lysate at 12 hpi, with an expected gradient of expression (Fig. 5A). This is consistent with the abundance of viral mRNA (Fig. 4A). In the IP of Rab11a, we identified high levels of NP, M, and C proteins (Fig. 5B). To define which proteins were specifically enriched by the Rab11a IP versus those just increasing in abundance after viral infection, we normalized the abundance of each protein identified in IP by its abundance in the whole-cell lysate. C proteins were the most highly enriched viral proteins in IP after normalization (Fig. 5C). Similar results were observed at 24 hpi, where all viral proteins were more abundant but with ranges similar those seen at 12 hpi (Fig. 5D to F). We also compared data representing the log_2_ fold change in the levels of proteins identified by IP at 12 hpi (Fig. 5G) and 24 hpi (Fig. 5H) and identified the C proteins as the most significantly enriched viral proteins interacting with Rab11a compared to mock-infected (mock) cells at both time points. Additionally, L protein was identified as significantly enriched at 24 hpi. To validate these results, we performed Western blot analysis and probed for Rab11a and various viral proteins (Fig. 5I). As expected, Rab11a was enriched after IP, while the viral protein P was not seen, corresponding to its absence in the mass spectrometry analysis. C protein had the highest ratio of IP to input, strongly suggesting that the C protein is the protein that most directly interacts with Rab11a. Both NP and M proteins were present in the IP at moderate levels, which likely reflects high levels of NP on the vRNP which may be coprecipitated and potential interactions between Rab11a and M which may occur at the plasma membrane in late stages of particle formation (see Fig. 1). Since SeV generates four C proteins that are described to have discrete functions (48), we asked whether we could identify which of the C proteins was interacting with Rab11a. The four C proteins range in size from 175 to 215 amino acids, and they share a 175-amino-acid C terminus. We mapped the peptide reads identified by mass spectrometry in the IP to the C proteins (Fig. 5J) and found they were all from the shared 175-amino-acid region. Therefore, we could not conclude which specific protein(s) was identified. Overall, the identification of C proteins as a likely interactor of Rab11a by immunoprecipitation and subsequent mass spectrometry supports results showing high degrees of colocalization as shown in Fig. 2 and suggests that C proteins play an important role in directing the interaction of vRNPs with recycling endosomes.

10.1128/mBio.02028-20.1 TABLE S1 Time course monitoring of changes in Rab11a-GFP-associated host and viral proteins during Sendai virus infection. The table data include averages of levels of protein abundance detected in three biological replicates for mock-, 12-hpi, and 24-hpi-infected cells. Fold change values were obtained for proteins identified at 12 hpi or 24 hpi compared to mock-infected cells; *t* test *P* values are indicated. “*Protein Accession*” refers to the UniProt database; “#*Peptides*” highlights the number of razor and unique peptides used for protein quantification; “*# Protein Unique Peptides*” highlights the number of peptide sequences unique for a given protein; “*Coverage [%]*” represents the percentage of the protein sequence covered by the peptides identified in the MS run; “*PSMs*” (peptide spectrum matches) show the total number of identified spectra assigned to peptide sequences for the protein, including those redundantly identified; *CV*, coefficient of variation; *Stdev*, standard deviation. For follow-up analysis, only proteins identified with a minimum of 2 peptides *(#Peptides*; razor and/or unique) were selected. Download Table S1, XLSX file, 0.3 MB. Copyright © 2020 Genoyer et al. 2020 Genoyer et al. This content is distributed under the terms of the Creative Commons Attribution 4.0 International license.

10.1128/mBio.02028-20.2 TABLE S2 Quantitative whole-cell proteome analysis of Sendai virus-infected cells. The table includes averages of levels of protein abundance detected in three biological replicates for mock-, 12-hpi, and 24-hpi-infected cells; fold change values were obtained for proteins identified at 12 hpi or 24 hpi compared to mock-infected cells; *t* test *P* values are indicated. “*Protein Accession*” refers to the UniProt database; “#*Peptides*” highlights the number of razor and unique peptides used for protein quantification; “*# Protein Unique Peptides*” highlights the number of peptide sequences unique for a given protein; “*Coverage [%]*” represents the percentage of the protein sequence covered by the peptides identified in the MS run; “*PSMs*” (peptide spectrum matches) show the total number of identified spectra assigned to peptide sequences for the protein, including those redundantly identified; *CV*, coefficient of variation; *Stdev*, standard deviation. Download Table S2, XLSX file, 1.6 MB. Copyright © 2020 Genoyer et al. 2020 Genoyer et al. This content is distributed under the terms of the Creative Commons Attribution 4.0 International license.

### Occupancy of polymerase proteins on viral genomes is a differentiating factor for interactions of vRNPs with Rab11a.

**FIG 6:**
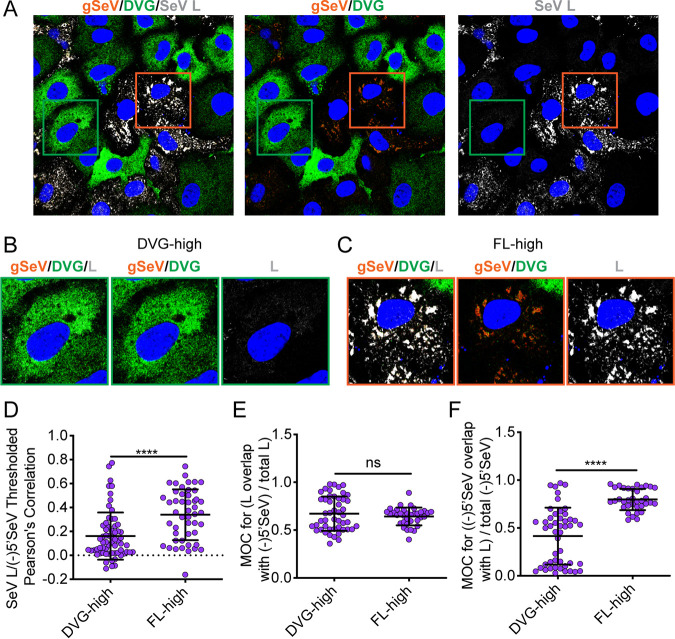
L protein interaction with vRNP distinguishes between DVG-high and FL-high cells. (A) A549 cells infected with SeV LeGFP supplemented with purified DPs (number of hemagglutinating units [HAU] = 20) for 24 h and then subjected to RNA FISH with immunofluorescence for GFP (L, gray) and Hoechst staining (nuclei, blue). Confocal 63×, 1.5× zoom, single-plane images are shown. Images are representative of results from four independent experiments; scale bar = 20 μm. Green boxes indicate representative DVG-high cells; orange boxes indicate representative FL-high cells. (B) Representative cropped DVG-high cell. (C) Representative cropped FL-high cell. (D) Costes’s Pearson’s correlation of colocalization between (-)5′ SeV and SeV NP in individual DVG-high and FL-high cells. (E) Mander’s overlap coefficient of L (GFP) with 5′ SeV in DVG-high and FL-high cells. (F) Mander’s overlap coefficient of 5′ SeV with L (GFP) in DVG-high and FL-high cells. Individual cells pooled from three independent experiments are plotted, with the horizontal dotted line at the mean; error bars represent SD. **** = *P* < 0.0001 by Mann-Whitney U-test.

Because we found that the level of L protein was lowest in DVG-high cells compared to FL-high cells and identified its cofactor C by mass spectrometry as a Rab11a interacting partner, we considered whether differences in polymerase component levels might drive differences in engagement with Rab11a in DVG-high and FL-high cells. To ask whether polymerase was associated with viral RNA in DVG-high cells, we performed RNA FISH combined with immunofluorescence. To visualize L, we used a recombinant SeV strain with the L protein C-terminally fused to enhanced GFP (SeV LeGFP) that had been previously described (43) and the addition of purified defective particles (pDPs) containing DVG-546, the dominant DVG in SeV Cantell. Previous studies have validated the ability of other strains of SeV to replicate DVGs from heterologous strains (41). During infection performed with SeV LeGFP in the presence of pDPs, we saw the establishment of DVG-high cells; strikingly, these cells had very low levels of L protein (Fig. 6A), recapitulating flow cytometry results that showed large differences in the amount of L protein in DVG-high cells (Fig. 6B) and FL-high cells (Fig. 6C). Additionally, it appears that a majority of the DVG RNA in DVG-high cells is not bound by L protein. Indeed, the level of colocalization of 5′(-)SeV RNA and L was significantly lower in DVG-high cells than in FL-high cells (Fig. 6D). The values corresponding to MOC of L were equivalent in DVG-high and FL-high cells, indicating that the same proportions of L in each cell were interacting with vRNA. The mean score nearing 1 indicates that the small amount of polymerase present in those cells colocalized with DVG RNA (Fig. 6E). However, the MOC of 5′(-)SeV RNA with SeV L was significantly lower in DVG-high cells compared to FL-high cells, indicating that a smaller fraction of viral RNA overlaps L in DVG-high cells than in FL-high cells and that there are many DVG RNAs that do not colocalize with detectable polymerase in DVG-high cells (Fig. 6F). The small amount of L protein in DVG-high cells is likely associated with DVGs for replication, but the majority of DVGs within the cytoplasm of DVG-high cells are not associated with detectable levels of L. This strong difference in L interaction with viral RNA in DVG-high and FL-high cells indicates that L protein may be critical in driving vRNPs to interact with Rab11a.

**FIG 7:**
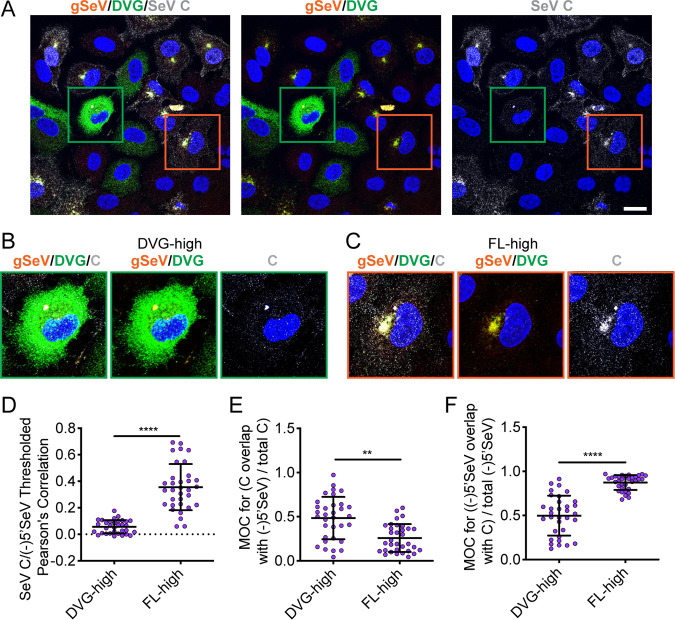
C protein interaction with vRNP distinguishes between DVG-high and FL-high cells. (A) A549 cells infected with SeV Cantell for 24 h and then subjected to RNA FISH with immunofluorescence for C protein (gray) and Hoechst staining (nuclei, blue). Confocal 63×, 1.5× zoom, single-plane images are shown. Images are representative of results from four independent experiments; scale bar = 20 μm. Green boxes indicate representative DVG-high cells; orange boxes indicate representative FL-high cells. (B) Representative cropped DVG-high cell. (C) Representative cropped FL-high cell. (D) Costes’s Pearson’s correlation of colocalization between (-)5′ SeV and SeV C in individual DVG-high and FL-high cells. (F) Mander’s overlap coefficient of C with 5′ SeV in DVG-high and FL-high cells. (E) Mander’s overlap coefficient of 5′ SeV with C protein in DVG-high and FL-high cells. Individual cells pooled from two independent experiments are plotted, with the horizontal dotted line at the mean; error bars represent SD. **** = *P* < 0.0001 by Mann-Whitney U-test.

Since engagement with the polymerase seems to be a differentiating factor between DVG-high and FL-high cells, we sought to investigate whether similar differences were found with C protein as well. Using RNA FISH combined with immunofluorescence for C protein, we observed similar results to those seen with SeV L, with low levels of C protein in DVG-high cells and a perinuclear localization of C protein in FL-high cells, as well as at the plasma membrane (Fig. 7A). These results suggest that at later time points of infection, C protein associates with vRNA and presumably L. Quantification of colocalization revealed that there is a significantly greater degree of colocalization of C and 5′(-)SeV RNA in FL-high cells (Fig. 7C) compared to DVG-high cells (Fig. 7B). This observation suggests that like with L protein, many DVG vRNPs are not occupied by C protein (Fig. 7D). MOC of SeV C reveals that higher proportions of C are colocalized with 5′(-)SeV RNA in DVG-high cells than in FL-high cells (Fig. 7E). However, MOC of 5′(-)SeV RNA with SeV C indicates that a high proportion of vRNA overlaps C in FL-high cells whereas much of the vRNA does not overlap C proteins in DVG-high cells (Fig. 7F). These data indicate that, similarly to L protein, low levels of C proteins in DVG-high cells seem to colocalize with DVG-RNA but that very low levels of C protein in the cell leave many RNAs unbound to free RNA. Taken together, these data suggest that lack of accumulation of C and L proteins in DVG-high cells and, therefore, lack of C and L proteins bound to vRNPs precludes these vRNPs from interacting with Rab11a.

## DISCUSSION

**FIG 8:**
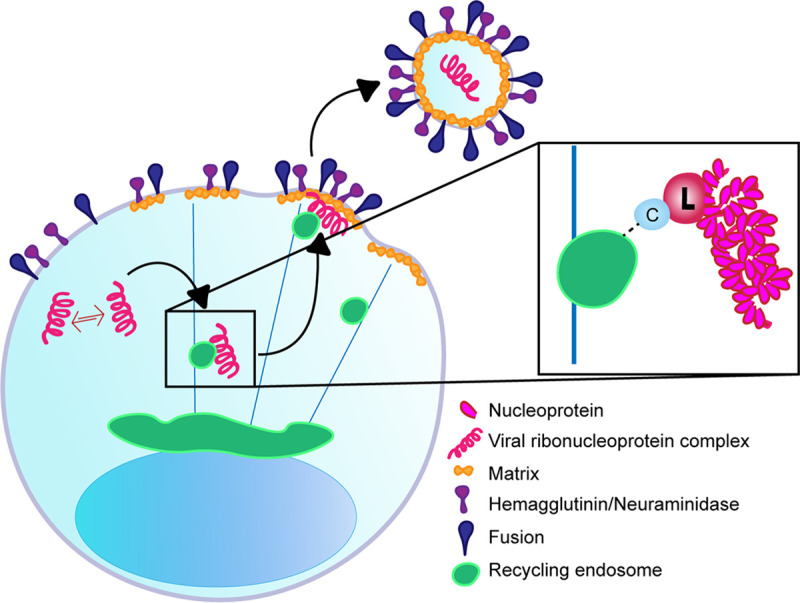
Model of SeV vRNP interaction with Rab11a. Viral replication occurs in the cytoplasm and upon accumulation of sufficient levels of L and C proteins; vRNPs interact with Rab11a-marked recycling endosomes via C and L proteins, to enable trafficking to the plasma membrane.

We propose a model of Sendai virus assembly in which polymerase components, including the viral polymerase L and its cofactor C, are critical for the interaction between viral RNPs and Rab11a. This interaction is a crucial initial step in viral particle production. In taking advantage of the differences in viral particle production in DVG-high and FL-high cells, we were able to parse the differences between viral components that are critical for replication and those that are critical for viral assembly. DVG-high cells are able to carry out robust levels of viral genome replication using limited amounts of L but are unable to produce virions because they do not engage with host proteins critical for this process (41). We observed that DVG interference with viral protein expression critically diminishes levels of viral polymerase L in DVG-high cells. We propose that L/C accumulation above a certain threshold is required for interaction of the vRNP with recycling endosomes and the cellular trafficking machinery. Furthermore, using mass spectrometry, we identified the C proteins as the most highly enriched viral proteins interacting with Rab11a. From these data, we propose that recycling endosomes interact with C proteins when it is associated with L and that C-L polymerase complexes interact with vRNPs to tether them to recycling endosomes (Fig. 8).

It remains to be determined whether paramyxoviruses interact directly with recycling endosomes via endosomal membrane interactions, with the Rab11a GTPase itself, with family-interacting proteins (FIPs) associated with Rab11a, or via another mechanism. The different Rab11a-FIPs direct engagement of Rab11a-marked recycling endosomes with dynein or myosin Vb motor proteins, and specific FIPs have been identified as important for budding of different viruses. For example, Rab11a-FIP2 is important for respiratory syncytial virus budding (49) and influenza virus relies on Rab11a-FIP3 for filamentous particle production (50). The Rab11a-FIPs used for trafficking of SeV are unknown, and identifying the viral protein that mediates interaction with recycling endosomes will aid further study of these dynamics.

Negative-sense RNA viruses require packaging of RdRPs within virions in order to initiate infection. Indeed, SeV L protein has been shown to associate with viral nucleocapsids isolated from virions (51). Additionally, C proteins have been found to be tightly associated with nucleocapsid structures both inside the cell and when isolated from SeV virions, with approximately 40 C proteins per vRNP (52). The observed roles of polymerase components in assembly are consistent with what was observed in infections with the orthomyxovirus influenza virus, where influenza virus RNPs interact with Rab11a via one of its polymerase components, PB2 (53, 54). As has been suggested for influenza virus, having the SeV polymerase complex initiate packaging via interactions with Rab11a could be a mechanism to ensure that only those vRNPs that contain a RdRP, and are thus infectious, can be packaged into virions.

Most paramyxoviruses encode a number of accessory proteins from the P gene, either through RNA editing leading to interferon antagonist V and W families or by translating alternative reading frames from the P mRNA (55) to generate C family members. While we propose that C proteins are critical for engagement with Rab11a, we suggest that in the absence of sufficient accumulation of L protein, vRNPs would be unable to interact with Rab11a. Thus, even if, for example, levels of C protein were higher in DVG-high cells, we would not expect to see C interacting with vRNPs in the absence of L. Additionally, while the four C proteins (C, C′, Y1, and Y2) have been previously described as having discrete functions in regulating pathogenesis and synthesis of mRNA and vRNA synthesis (48), we are unable to distinguish from our results whether a specific C protein is required for interaction with Rab11a or if they are interchangeable. The specific C proteins required for SeV assembly and whether C family proteins of other paramyxoviruses play similar or distinct roles in viral assembly are as yet unknown. Discerning their function in assembly could provide specific targets for development of directly acting antivirals.

We also investigated the role of SeV M in viral assembly. M lines the inner side of virions and interacts directly with nucleoproteins (23, 56). These contacts between M and the nucleoproteins drive virus-specific viral particle production, since studies combining M and nucleoproteins from the closely related SeV and HPIV1 failed to lead to particle production (57). While M-NP contacts are important for particle production, it is unknown whether M is delivered to the cell membrane with nucleoproteins or independently and whether its transport relies on Rab11a (14). Our experiments indicate that M traffics independently to the plasma membrane and is not the key mediator in vRNP interactions with Rab11a. Our data are supported by previous reports that indicate that M traffics with F and HN through the trans-Golgi network (TGN) to the cell membrane (24). Although we identified M in the Rab11a IP-mass spectrometry data, this result could not be validated by Western blotting. This may reflect interactions between M and Rab11a occurring at the plasma membrane, at the TGN or the endoplasmic reticulum (ER). If M is trafficked to the membrane with surface proteins F and HN as proposed previously (24), Rab11a may be interacting with M near the TGN. There is evidence that the rhabdovirus vesicular stomatitis virus (VSV) glycoprotein, which is processed in the TGN, requires Rab11a for proper sorting to the plasma membrane (58). There is also evidence that Rab11a is recruited to the ER during influenza virus infection to facilitate transport of vRNPs (59). Therefore, it is possible that SeV M, F, and HN interact with Rab11a during their transit from the ER through the TGN to the plasma membrane.

It is well established that DVGs exert effects on viral infections through interfering with standard viral genomes by competing for structural proteins or viral polymerase (29). DVGs have been reported to reduce levels of viral proteins globally within infected cells and for that reason have been investigated as possible antivirals themselves (60). Whether all viral proteins are equally affected by interference has not been previously determined. While it was previously shown that infections with SeV HD lead to lower levels of M protein, these lower levels were attributed to increased degradation of M (42). The presence of DVGs was also previously shown to lead to lower levels of HN on the surface of infected cells (61). We performed a systematic investigation of the effects of interference on levels of mRNA and protein levels by directly comparing FL-high cells and DVG-high cells from a heterogenous infection. Our results support previous findings of lower levels of M and HN in the presence of DVGs but reveal that DVG interference with L mRNA transcription leads to significantly smaller amounts of mRNA and subsequent lower levels of protein translation in DVG-high cells than have been seen with other viral proteins. Since L is the lowest-abundance mRNA and protein, DVG-driven interference appears to have the highest impact on its abundance. The gradient of expression of mRNA and the generation of copy-back DVGs are unique to single-stranded negative-sense RNA viruses. However, other RNA viruses, such as influenza virus, produce deletion DVGs and are known to be sensitive to DVG-driven interference. It was previously shown that DVGs generated from certain segments can exert different levels of interference pressure (62) but not whether interference particularly affects the accumulation of certain mRNAs or proteins. The role that DVG-mediated interference may play in restricting viral progeny production via reduction of certain key proteins in other viral families remains to be investigated.

Overall, using DVGs as a tool to understand differences in viral replication and viral assembly, we have shown that polymerase cofactors may be critical in inducing primary steps of viral particle production. While we propose that C and L proteins are essential for driving contact between vRNPs and Rab11a during SeV infection, whether intricacies of viral assembly are conserved across paramyxoviruses is unknown. Many studies suggest that there are at least subtle differences among the viruses in their intracellular localization, transport, and eventual assembly (14). Because DVGs have been identified from most paramyxoviruses, comparisons between DVG-high and FL-high cells during infection may generally be useful in differentiating the proteins required for viral replication from those required to initiate virion assembly of other members of the viral family.

## MATERIALS AND METHODS

### Cells.

A549 (human type II pneumocytes, ATCC CCL-185), HEK 293T/17 (ATCC CRL-11268), and LLCMK2 (monkey kidney epithelial cells, ATTC CCL-7) cells were maintained in tissue culture medium (Dulbecco’s modified Eagle’s medium [DMEM; Invitrogen] supplemented with 10% fetal bovine serum [FBS; Sigma], gentamicin [Thermo Fisher], sodium pyruvate [Invitrogen], and l-glutamine [Invitrogen]) at 7% CO_2_ and 37°C. A549 Rab11a-GFP and A549 Rab11a-mCherry cells were generated as described previously (54). Cells were tested monthly for mycoplasma by the use of a MycoAlert Mycoplasma detection kit (Lonza) and treated with mycoplasma removal agent (MP Biomedical) upon thawing.

### Viruses.

Sendai virus Cantell was grown in 10-day embryonated specific-pathogen-free chicken eggs (Charles River) for 40 h before allantoic fluid was collected. Low-DVG and high-DVG stocks were produced as previously described (38). Defective particles (DPs) containing DVG-546 were purified from allantoic fluid infected with SeV Cantell by density ultracentrifugation on a 5% to 45% sucrose gradient, using 50% tissue culture infective dose (TCID_50_) data to confirm the absence of virus and direct hemagglutination to detect particles (34). SeV-LeGFP is a recombinant Enders strain of SeV with eGFP fused to the C terminus of the L protein as described previously (43). SeV-LeGFP cells was grown for 72 h in LLCMK2 cells. SeV-M-HA virus was generated with an HA tag at the N terminus of the M protein in a recombinant SeV F1R background with an eGFP reporter. Virus was rescued by transfection into BSRT7 cells as described previously (63) and passaged in a blind manner three times. Virus was then grown for 5 days in LLCMK2 cells. All viruses were titrated using 1:10 serial dilution in triplicate in LLCMK2 cells in the presence of 2 μg/ml tosylsulfonyl phenylalanyl chloromethyl ketone (TPCK)-treated trypsin (Worthington Biomedical) for 72 h, and TCID_50_/ml levels were determined by the Reed-Muench method based on hemagglutination of 0.5% washed chicken red blood cells (Lampire) by supernatant to detect the presence of infection.

### Plasmids and transfection.

SeV-M-FLAG plasmid encodes codon-optimized SeV matrix protein with a 3× N-terminal FLAG tag in the pCMV-3Tag-1A vector. Cells in antibiotic-free media (8 × 10^5^ cells/well of a 6-well plate) were transfected 6 h postinfection with 2 μg plasmid and 6 μl Lipofectamine 2000 (Invitrogen) per well diluted in Opti-MEM (Invitrogen).

### Viral infections.

Infections were performed at a multiplicity of infection of 1.5 TCID_50_/cell unless otherwise specified. Cells were seeded to reach 80% confluence on the day of infection (4 × 10^5^ A549/well in 6-well plates). Prior to infection, cells were washed twice with phosphate-buffered saline (PBS). Virus was diluted in infection media (DMEM [Invitrogen] supplemented with 35% bovine serum albumin [BSA; Sigma-Aldrich], penicillin/streptomycin [Pen/Strep; Invitrogen], and 5% NaHCO_3_ [Sigma-Aldrich]), and a low volume of inoculum was added to cells for 1 h of incubation at 37°C, with shaking every 15 min. Cells were then replenished with 2% FBS tissue culture medium. Viral infections were performed in the presence of pDPs by adding pDPs at a dilution of 20 direct hemagglutination units/4 × 10^5^ cells along with virus.

### Drug treatment.

Four hours postinfection, the medium was removed and replaced with 2% FBS tissue culture media containing 2 μg/ml Nocodazole (Sigma-Aldrich) for the duration of infection.

### Immunofluorescence.

Cells were seeded on no. 1.5 glass coverslips (Corning) overnight prior to infection. After infection, coverslips were rinsed in PBS and then fixed for 10 min in 4% paraformaldehyde–PBS (Electron Microscopy Sciences) for 10 min. Cells were permeabilized with 0.2% Triton X–PBS (Sigma-Aldrich) for 10 min. Primary and secondary antibodies were diluted in 3% FBS–PBS and incubated at room temperature for 1.5 h and 1 h, respectively. Nuclei were stained with Hoechst stain prior to mounting of coverslips on slides using Fluoromount-G (Thermo Fisher). The primary antibodies used were as follows: mouse anti-SeV NP (clone M73/2 [a kind gift from Alan Portner] directly conjugated with DyLight 594 N-hydroxysuccinimide [NHS] ester [Thermo Fisher]), rabbit anti-HA tag (CST C29F4), mouse anti-FLAG 9A3 (CST 8146), rabbit anti-GFP (ab6556), mouse anti-C (clone P16, 96-6 [from Toru Takimoto]). The secondary antibodies used were as follows: goat anti-rabbit IgG (H+L) secondary antibody, Alexa Fluor 488 (Invitrogen); goat anti-mouse IgG (H+L) secondary antibody, Alexa Fluor 647 (Invitrogen); goat anti-mouse IgG (H+L) secondary antibody, Alexa Fluor 488 (Invitrogen).

### RNA FISH.

Custom probe sets were designed against the SeV genome (described in reference 41) and conjugated to Quasar 570 [3′(-)SeV] and Quasar 670 [5′(-)SeV] dyes (LGC Biosearch). For RNA FISH microscopy, cells were seeded on no. 1.5 glass coverslips (Corning) prior to infection. After infection, the coverslips were rinsed with PBS and then fixed in 3.7% formaldehyde (Thermo Fisher) for 10 min. The cells were permeabilized in 70% ethyl alcohol (EtOH) for 1 h at room temperature and then washed in wash buffer (2× SSC [Thermo Fisher] [1× SSC is 0.15 M NaCl plus 0.015 M sodium citrate] and 10% formamide [Thermo Fisher] in nuclease-free water) and subjected to hybridization with FISH probes. For hybridization, probes were diluted to 2.5 nM in hybridization buffer (wash buffer plus dextran sulfate) and applied to slides. Slides were incubated with probe overnight at 37°C in a humidified chamber. Prior to imaging, slides were washed in wash buffer twice (once with Hoechst to stain nuclei) and then 2× SSC. Cells were mounted in ProLong Diamond antifade mountant (Thermo Fisher) and cured overnight at room temperature prior to imaging. RNA FISH combined with immunofluorescence was modified by staining cells with antibody after permeabilization with EtOH. Cells are stained with antibody–1% BSA (Thermo Fisher)–PBS–RNaseOUT (Invitrogen) for 45 min for primary Ab and 40 min for secondary Ab. Cells were then postfixed for 10 min in 3.7% formaldehyde prior to hybridization.

### RNA FISH flow cytometry.

Cells were harvested postinfection by trypsinization (Thermo Fisher) and centrifugation. Cell pellets were washed in 1% FBS–PBS one time. Cell pellets were then resuspended in 10 ml ice-cold methanol (Thermo Fisher) and fixed and permeabilized on ice for 10 min before pelleting was performed at 1,500 rpm for 5 min. Pellets were then resuspended in 1 ml wash buffer per sample and transferred to a microcentrifuge tube, where they were washed twice. For hybridization, probes were diluted to 25 nM in 100 μl hybridization buffer (wash buffer plus dextran sulfate) and cell pellets were resuspended in hybridization buffers and incubated overnight at 37°C. For antibody staining, primary antibodies were added to the hybridization buffer and incubated overnight. After hybridization, cell pellets were washed in wash buffer twice and then washed in 2× SSC before being resuspended in anti-fade buffer (2× SSC, 0.4% glucose [Sigma], and Tris-HCl [pH 8.0; USB Corporation] with catalase [Sigma] and glucose oxidase [Sigma]). For secondary antibody staining, antibody was added to wash buffer prior to washing with 2× SSC and the reaction mixture was incubated at 37°C for 30 min. The primary antibodies used were as follows: mouse anti-SeV NP (clone M73/2; a kind gift from Alan Portner), mouse anti-SeV F (clone 11H12), mouse anti-SeV HN (clone 1A6), rabbit anti-HA tag (CST C29F4), and rabbit anti-GFP (ab6556). The secondary antibodies used were as follows: goat anti-rabbit IgG (H+L) secondary antibody, Alexa Fluor 405 (Invitrogen); goat anti-mouse IgG (H+L) secondary antibody, Alexa Fluor 405 (Invitrogen). Flow cytometry was performed on a BD LSRFortessa cell analyzer, and compensation and gating were performed in FlowJo_v9.9.4 (BD Biosciences). Mock and SeV LD infections were used to determine gating of SeV HD DVG-high and FL-high populations.

### Microscopy and image analysis.

Images were acquired on a Leica SP5-II laser scanning confocal microscope with 63× (1.40 to 0.60 numerical aperture [NA]) and 100× (1.46 NA) oil-immersion lens objectives with pixel sizes of 70 nm by 70 nm for FISH immunofluorescence, with a z-step size of 0.13 μm, with 15 slices in total, and with a 2,048-pixel-by-2,048-pixel image format. Immunofluorescence images (Rab11a colocalization) were acquired with the same parameters except for a pixel size of 40 nm by 40 nm. Images were processed in Volocity (Perkins-Elmer), and deconvolution, when performed, was done with Huygen’s Essential Deconvolution Wizard using theoretical point spread function. Colocalization was quantified in Volocity over total cell volume with manually defined regions of interest (ROIs) using automatic thresholding based on a previously published method (64), and cells were binned as DVG-high or FL-high based on the ratio of 3′(-)SeV to 5′(-)SeV.

### Immunoprecipitation.

Cells were lysed in NP-40 lysis buffer (Amresco) with proteinase inhibitors (Roche Boehringer Mannheim), RNase OUT (Invitrogen), and EDTA (Thermo Fisher). Protein concentrations were measured using bicinchoninic acid (BCA) protein assay (Thermo Fisher). For anti-NP immunoprecipitation assay, 500 μg of protein was incubated overnight with primary antibody (anti-SeV NP). Protein G magnetic beads (EMD Millipore (30 μl per sample) were blocked overnight with salmon sperm DNA (Thermo Fisher)–5% FBS–PBS. Beads were washed and incubated with lysate/antibody for 4 h at 4°C with rotation. For anti-NP immunoprecipitations, beads were added three times sequentially. Beads were then washed with high-salt lysis buffer three times then with low-salt lysis buffer once. Processing of samples for RNase treatment was stopped at that point; samples for other applications were boiled in sample buffer for 10 min. For Rab11a-GFP immunoprecipitation, anti-GFP immunoprecipitation was carried out using anti-GFP monoclonal antibody (MAb) magnetic beads. Lysates (500 μg) were incubated overnight with beads and then washed five times with wash buffer (50 mM Tris-HCl [pH 7.5], 150 mM NaCl, 0.05% NP-40).

### Sample preparation for proteomic analysis.

All chemicals used for preparation of nanoflow liquid chromatography-tandem mass spectrometry (nLC-MS/MS) samples were of sequencing grade and were purchased from Sigma-Aldrich (St. Louis, MO), unless otherwise stated. Immunoprecipitated Rab11a-GFP interacting proteins were eluted from the magnetic beads by the use of the on-bead tryptic digestion method. Briefly, the beads were resuspended in 50 μl of 50 mM trimethylammonium bicarbonate (TEAB; Thermo Fisher Scientific, Waltham, MA) (pH 8), and proteins were reduced using 10 mM dithiothreitol (DTT) for 1 h at room temperature and alkylated with 20 mM iodoacetamide (IAA) in the dark for 30 min at room temperature. Proteins were digested with trypsin (Promega, Madison, WI) at an enzyme-to-substrate ratio of ∼1:50 for 12 h in a thermomixer, with shaking at 900 rpm, at room temperature. After digestion, the supernatant was removed and collected into fresh, labeled tubes. Beads were washed twice with 50 μl of the wash buffer (50 mM TEAB [pH 8.5], 5% acetonitrile), and all of the supernatants were merged. The samples were concentrated to the volume of ∼100 μl by lyophilization and acetified with trifluoroacetic acid (TFA) to a final concentration of 0.1%. The tryptic peptides were desalted using Poros Oligo R3 RP (PerSeptive Biosystems, Framingham, MA) P200 columns with a C_18_ 3 M plug (3 M Bioanalytical Technologies, St. Paul, MN) prior to nLC-MS/MS analysis.

The whole-cell proteome samples were processed using the suspension trap (S-Trap; Protifi, Huntington, NY) (65) mini-spin column digestion protocol with minor modifications. Briefly, cells were lysed in 300 μl of lysis buffer (5% SDS, 50 mM TEAB [pH 7.55], Halt protease and phosphatase inhibitor cocktail [Thermo Fisher Scientific, Waltham, MA]) by vortex mixing and probe tip sonication at 4°C. The lysate was clarified by centrifugation at 13,000 × *g* for 10 min at 4°C. Protein concentrations were measured by Bradford protein assay (Thermo Fisher Scientific), and ∼300 μg of reduced and alkylated proteins was subjected to trypsin digestion following the procedure described by the manufacturer of the S-Trap. The peptide solutions were pooled, lyophilized, and desalted prior to nLC-MS/MS.

### Nanoflow liquid chromatography tandem mass spectrometry (nLC-MS/MS).

The peptide mixture was separated using a Dionex Ultimate 3000 high-performance liquid chromatography (HPLC) system (Thermo Fisher Scientific) with a two-column setup, consisting of a reversed-phase trap column (Acclaim PepMap100 C_18_; Thermo Fisher Scientific) (5-μm pore size, 100 Å, 300-μm inner diameter [i.d.] by 5 mm) and a reversed-phase analytical column (30 cm, 75-μm i.d., 360 μm o.d., packed in-house with Pur C18AQ [Dr Maisch; 3-μm pore size]). The loading buffer used was 0.1% trifluoroacetic acid (Merck Millipore)–water. Buffer A was 0.1% formic acid, and buffer B was 80% acetonitrile plus 0.1% formic acid. The HPLC system was coupled online with an Orbitrap Fusion mass spectrometer (Thermo Fisher Scientific, San Jose, CA). The gradients were 135 min from 2% to 36% buffer B at a flow rate of 300 nl/min for Rab11a-GFP interacting protein samples and 180 min for whole-cell proteome samples. The MS instrument was controlled by Xcalibur software (Thermo Fisher Scientific). The nanoelectrospray ion source (Thermo Fisher Scientific) was used with a spray voltage of 2.2 kV. The ion transfer tube temperature was 275°C. Data acquisition was performed in the Orbitrap mass spectrometer for precursor ions. MS survey scans were obtained for the *m*/*z* range of 350 to 1,200 in the Orbitrap mass spectrometer with a maximum ion injection time of 100 ms, an automatic gain control target of 5 × 10^5^, and a mass resolution value of 120,000. MS/MS was performed with the TopSpeed duty cycle set to 3 s. Dynamic exclusion was set to 4 s. The charge state range enabled was 2^+^ to 6^+^. The value for higher collisional dissociation (HCD) was set to 30. MS/MS data were acquired in the ion trap using the Rapid scan mode, automatic gain control set to 10,000, and maximum injection time set to 120 ms.

### Identification and quantification of proteins.

The raw mass spectrometer files were processed for protein identification using Proteome Discoverer (v2.4; Thermo Fisher Scientific) and the Sequest HT algorithm with a peptide mass tolerance of 10 ppm, fragment *m*/*z* tolerance of 0.25 Da, and a false-discovery rate (FDR) of 1% for proteins and peptides. All peak lists were searched against the UniProtKB/Swiss-Prot database of human sequences (January 2020; 20,367 entries) and the UniProtKB/TrEMBL database of Sendai virus (Cantell) sequences (February 2020; 8 entries) using following parameters: enzyme, trypsin; maximum number of missed cleavages, 2; fixed modification, carbamidomethylation (C); variable modifications, oxidation (M) and protein N terminus acetylation. Protein quantifications were log_2_ transformed and normalized using the median of the distribution for each sample. In order to estimate enrichment of proteins detected only in mock treatment or in infection, missing values were imputed using a distribution of values of 30% width and 2 standard deviations lower than the average of the distribution of valid values. Statistical analyses were performed on three different biological replicates. The sample size was chosen to provide enough statistical power to apply parametric tests (either homoscedastic test or heteroscedastic one-tailed *t* test, depending on the statistical value of the F-test; heteroscedastic for F-test *P* values of <0.05). The *t* test was considered a valuable statistical test because binary comparisons were performed and because the number of replicates was limited. No samples were excluded as outliers (this applies to all proteomics analyses described in this article). Proteins with a *t* test *P* value below 0.05 were considered to represent a significantly alteration between the two tested conditions. The data distribution was assumed to be normal, but this was not formally tested.

### Western blotting.

Whole-cell lysate (10 μg) or immunoprecipitation products were denatured by boiling for 10 min and run on a 4% to 12% gradient Bis-Tris gel (Bio-Rad) before being transferred to a polyvinylidene difluoride (PVDF) membrane (Millipore). Membranes were incubated overnight with primary antibody in 5% milk. Membranes were incubated with secondary antibody for an hour in 5% milk and developed using Lumi-light Western blot substrate (Roche) to detect horseradish peroxidase (HRP). Images were acquired using a ChemiDoc Bioimager (Bio-Rad) and quantified in ImageJ. The primary antibodies used were as follows: rabbit anti-Rab11a (Invitrogen); mouse anti-C (clone p96-6; a kind gift from Toru Takimoto). SeV NP, P, and M were detected by the use of chicken anti-SeV polyclonal antibody (Abcam). The secondary antibodies used were as follows: anti-rabbit HRP-conjugated antibody (Cell Signaling), anti-mouse IgG for IP (Abcam), and anti-chicken HRP-conjugated antibody (Invitrogen).

### RNase treatment.

Postimmunoprecipitation unbound fractions or substrate bound to magnetic beads was subjected to treatment with a combination of 1 U/ml RNase A, V, and T1 (Invitrogen) for 15 min at room temperature. RNase reactions were stopped by adding TRIzol LS (Invitrogen) to the sample.

### RNA extraction and RT-qPCR.

Cellular and viral RNA was harvested using TRIzol (Invitrogen). RNA was reverse transcribed using a SuperScript III first-strand synthesis system (Invitrogen) with Oligo(dT) for mRNA-specific amplification or with primer 5′-GGTGAGGAATCTATACGTTATAC-3′ for viral RNA. qPCR was performed with 1:40 dilution of cDNA, SYBR green (Life Technologies), and 5 μM forward/reverse primers (Invitrogen) on an Applied Biosystems ViiA 7 real-time system. Relative copy numbers per cell were calculated by the delta-delta threshold cycle (ΔΔ*C_T_*) method and normalized to average cellular GAPDH expression levels. Primer sequences are shown in parentheses as follows: SeV NP (F: 5′-TGCCCTGGAAGATGAGTTAG-3′; R: 5′-GCCTGTTGGTTTGTGGTAAG-3′); SeV P/C (F: 5′-GGATATCCGAGATCAGGTATTGA-3′; R: 5′-GGCCCGGTGTATATTTTGTTT-3′); SeV M F: (5′-GCCATCCCCTACATCAGGAT-3′; R: 5′-GTAACGACCCGGAGCCGCAT-3′); SeV F (F: 5′-CTCCTGAAGATCTCTAAGGCAT-3′; R: 5′-GGATCCCACGAATCGAGGTA3’); SeV HN F: (5′-GACCAGGAAATAAAGAGTGCA-3′; R: 5′-CGATGTATTGGCATATAGCGT-3′); SeV L (F: 5′-TGGTCAGAGATGCAACGAGA-3′; R: 5′-ACCTTTCAAGGACTGGATGC-3′); gSeV (F: 5′-GACCAGGAAATAAAGAGTGCA-3′, R: 5′-CGATGTATTGGCATATAGCGT-3′); DVG-546 (F: 5′-TCCAAGACTATCTTTATCTATGTCC-3′, R: 5′-GGTGAGGAATCTATACGTTATAC-3′).

### Data availability.

The mass spectrometry proteomics data have been deposited into the ProteomeXchange Consortium via the PRIDE (66) partner repository with the data set identifiers PXD019637 and https://doi.org/10.6019/PXD019637.
